# Hypofibrinogenemia Following an Anaphylactic Shock Caused by a Neuromuscular Blocking Agent—Case Report

**DOI:** 10.1155/crcc/6954832

**Published:** 2025-08-27

**Authors:** Michael Iarossi, Caroline van de Wyngaert, Véronique Hamoir, Geoffroy Vanderweerden, Cedric Hermans

**Affiliations:** ^1^ Division of Haematology, Haemostasis and Thrombosis Unit, Saint-Luc university Hospital, Université catholique de Louvain (UCLouvain), Brussels, Belgium, saintluc.be; ^2^ Laboratory Department, Centre Hospitalier de Wallonie Picarde (CHwapi), Tournai, Belgium; ^3^ Department of Anesthesiology, Centre Hospitalier de Wallonie Picarde (CHwapi), Tournai, Belgium

**Keywords:** anaphylaxis, case report, hypofibrinogenemia, muscle relaxants, rocuronium

## Abstract

Anaphylaxis, the most severe form of allergic reaction, has a prevalence of 1/5000–1/20,000 following general anesthesia. Numerous substances used in anesthesia, such as induction agents and muscle relaxants, can potentially trigger anaphylactic reactions. Muscle relaxants, particularly rocuronium (a nondepolarizing aminosteroid curare), are among the most frequently implicated agents. Anaphylaxis should be suspected when sudden‐onset symptoms affecting multiple systems manifest, including typical skin lesions, severe respiratory, cardiovascular, and/or gastrointestinal symptoms. In addition to its well‐known symptomatology, anaphylaxis may also induce less well‐described alterations in the coagulation system. Although cases of hyperfibrinolysis and disseminated intravascular coagulation have been reported, their incidence and clinical relevance remain unclear. Assessment of potential coagulation disorders related to anaphylaxis should involve both static blood tests specific to the coagulation pathways (e.g., INR, PTT, and fibrinogen) and viscoelastic coagulation tests (e.g., thromboelectography). Here, we present a rare case of a patient who experienced a significant anaphylactic reaction accompanied by hypofibrinogenemia following the administration of rocuronium during general anesthesia.

## 1. Introduction

Allergic reactions can be mediated by various mechanisms, including IgE, IgG, complement, immune complex‐mediated pathways, or nonimmune processes. Immune‐mediated allergic reactions involve the immediate release of physiologically active substances by mast cells (MCs) and basophils, making them key players in IgE‐mediated human allergic responses [1]. Among allergic reactions, anaphylaxis represents the most severe form. The incidence of allergic reactions to drugs is estimated to be around 1%–3% of exposures, but anaphylaxis occurs less frequently, with a prevalence ranging from 1 in 5000 to 1 in 20,000 cases during general anesthesia [2, 3]. Anaphylaxis is a rare yet significant event that can lead to substantial morbidity and mortality. It stands as the most common cause of complications during anesthesia, irrespective of the type of surgery, anesthetic management, or pre‐existing comorbidities [4].

Anaphylaxis can be triggered by a diverse range of substances utilized during anesthesia, such as induction agents, muscle relaxants, opioids, antibiotics, colloidal solutions, and blood products. Among these, muscle relaxants, specifically rocuronium (a nondepolarizing aminosteroid curare), are the most commonly implicated drugs in anaphylaxis cases [5]. Allergic reactions in the context of anesthesia are predominantly mediated by IgE antibodies. Surprisingly, up to 75% of these reactions have been reported to occur upon initial exposure, indicating a potential cross‐reactivity with IgE antibodies generated from previous contact with seemingly unrelated chemicals [4].

Anaphylaxis should be considered in any acute presentation involving multiple body systems, characterized by the presence of typical skin lesions such as urticarial rash, erythema, and/or angioedema, along with persistent and severe respiratory, cardiovascular, and/or gastrointestinal symptoms. Early recognition of signs and symptoms is crucial, followed by the prompt discontinuation or antagonization of the suspected triggering substance. Adrenaline is universally recommended as the first‐line treatment in all national guidelines for anaphylaxis management. Supplementary treatment options include the administration of corticosteroids and antihistamines, although the evidence supporting their use lacks high‐quality research. Furthermore, initiating hemodynamic support is essential, and airway clearance measures should be implemented if obstruction is present [6].

In addition to its well‐known impact on various body systems, anaphylaxis also affects the hemostasis system [7]. However, these hemostatic changes are often underreported and thus remain understudied, likely due to limited availability of viscoelastic coagulation tests (thromboelastography (TEG) or rotational thromboelastometry (ROTEM)) in acute settings and the primary focus of clinicians on life‐saving interventions. Nonetheless, a few cases of hyperfibrinolysis and disseminated intravascular coagulation (DIC) during anaphylaxis have been reported in the literature, notably demonstrated with the use of TEG [8]. Here, we present a rare case of a patient who experienced a significant anaphylactic reaction associated with an isolated hypofibrinogenemia following the administration of rocuronium during general anesthesia.

## 2. Case Description

**Figure 1:**
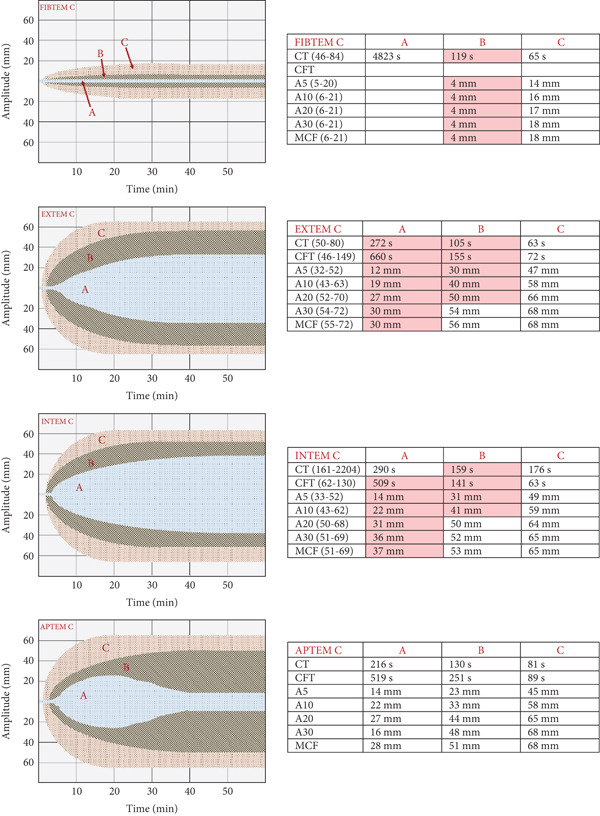
ROTEM performed on whole blood post anaphylaxis (A), post administration of fibrinogen (B) and 18 h postanaphylaxis (C). A: postanaphylaxis; B: postadministration of fibrinogen; C: 18 h postanaphylaxis; CT: clotting time; CFT: clot formation time; A5: amplitude 5 min after CT; A10: amplitude 10 min after CT; A20: amplitude 20 min after CT; A30: amplitude 30 min after CT; MCF: maximum clot firmness.

A 47‐year‐old male with a history of untreated allergic asthma was admitted to the hospital for a scheduled arthroscopy of the temporomandibular joint under general anesthesia. Apart from asthma, he had no significant medical history and did not take regular medications. This was his first general anesthesia. There was no known drug allergy and no personal or family history of bleeding disorders. The preoperative clinical and laboratory assessments showed no abnormal findings (Table 1). Anesthesia was initiated with propofol (200 mg), midazolam (8 mg), and rocuronium (40 mg). Following the administration of rocuronium, the patient experienced a sudden drop in blood pressure (89/53 mmHg), increased heart rate (142 bpm), and bronchospasm, leading to suspicion of an allergic reaction. As a result, the procedure was promptly halted. The physical examination revealed no abnormality except for the presence of bronchospasm, and there were no indications of abnormal bleeding or thrombotic events. An immediate blood test was conducted, which showed an elevated tryptase level of 43.6 *μ*g/L (normal values: < 8.23 *μ*g/L). The complement level and C1‐esterase inhibitor were within normal ranges. However, the coagulation parameters were severely disrupted, with a prolonged activated partial thromboplastin time (aPTT) of 49 s (normal range: 25–36.5 s), decreased fibrinogen level of 60 mg/dL (normal range: 150–450 mg/dL), elevated D‐dimer level exceeding 10,000 ng/mL (normal range: < 500 ng/mL), and a normal platelet count (Table 1). The ROTEM confirmed a coagulopathy with isolated hypofibrinogenemia (Figure 1). The initial management approach involved discontinuation of the procedure and immediate administration of the following medications: ephedrine (18 mg), sugammadex (a rocuronium antagonist; dose of 600 mg), adrenalin (1 mg), tranexamic acid (1000 mg), and hydrocortisone (100 mg).

**Table 1:** Evolution of biological parameters.

	**Normal values**	**Before surgery**	**Just after rocuronium**	**1 day after rocuronium**	**2 days after rocuronium**	**1 month after rocuronium**
Hb (g/dL)	13–17	15.6	14.9	13.2	11.1	13.6
Platelets (/mm^3^)	150,000–400,000	210,000	297,000	310,000	208,000	435,000
White blood cell count (/mm^3^)	4000–10,000	8150	25,730	30,260	14,050	8200
APTT^a^ (s)	20–30	25	49	23.6	38.3	30
PT^b^ (%)	75–100	94	10	94	> 100	
INR^c^		1	5.4	1	1	0.99
Fibrinogen (mg/dL)	200–400	230	60	224	358	369
D dimer (ng/mL)	< 500		> 10,000	> 10,000		
Factor V (%)	60–120		13			
Tryptase (*μ*g/L)	< 8.23		43.60			4.20

^a^Activated partial thromboplastin clotting time.

^b^Prothrombine time.

^c^International normalized ratio.

In light of the observed coagulation abnormalities and the absence of clinical signs of DIC such as bleeding or thrombosis, treatment was initiated with tranexamic acid (1000 mg, three times a day) and fibrinogen supplementation (4 g in total). The patient demonstrated rapid improvement both clinically and in laboratory tests (including normalization of ROTEM at 18 h), leading to discharge after 48 h of close monitoring in the intensive care unit. A comprehensive evaluation, performed at a later stage following the acute episode, included a thorough assessment of hemostasis and allergic markers. The assessment of primary hemostasis, coagulation, and fibrinolysis (Euglobulin lysis time) revealed no abnormality. Furthermore, the blood levels of tryptase returned to normal levels. Allergic skin tests performed for the different agents potentially implicated in the anaphylactic reaction were positive only for rocuronium. The diagnosis of anaphylactic shock caused by this specific substance was established based on the temporality, the clinical signs, the biological test results, and their normalization over time, as well as the positive skin tests.

## 3. Discussion

The link between anaphylaxis and coagulation disorders is well established, although not well known to physicians. Initially observed in animal studies [7, 9], this association has been further supported by various case reports. The potential consequences include DIC [10, 11] or primary hyperfibrinolysis [12]. While primary fibrinolysis has been documented in this context, the precise incidence and clinical significance of its occurrence remain uncertain [13]. This case report stands as one of the few recent studies that shed light on the interaction between acute coagulation disorders and anaphylaxis triggered by rocuronium, characterized by enhanced fibrinolytic‐type DIC [14].

Primary fibrinolysis is characterized by an absolute quantitative or qualitative abnormality of proteins directly involved in the lytic process, leading to widespread degradation of fibrinogen. On the other hand, secondary fibrinolysis can be applied to pathological situations when the members of the fibrinolytic system have normal structure and availability, but they either act on fibrin that is more susceptible to lysis or their hyperfunction is provoked in response to overt abnormal systemic blood clotting [15]. It is crucial to differentiate between primary and secondary conditions, as they represent distinct pathologies with varying prognoses and treatment approaches [16]. Indirect measures, such as platelet count, schistocyte count, and D‐dimer assay, are commonly employed to assess fibrinolysis. However, viscoelastic coagulation tests, such as ROTEM and TEG, which can provide real‐time recognition of hyperfibrinolysis, are rarely reported in case studies, mainly due to their limited availability. Nevertheless, these advanced tests offer valuable insights and can serve as valuable tools in guiding the management and treatment of fibrinolysis‐related conditions [17].

**Figure 2:**
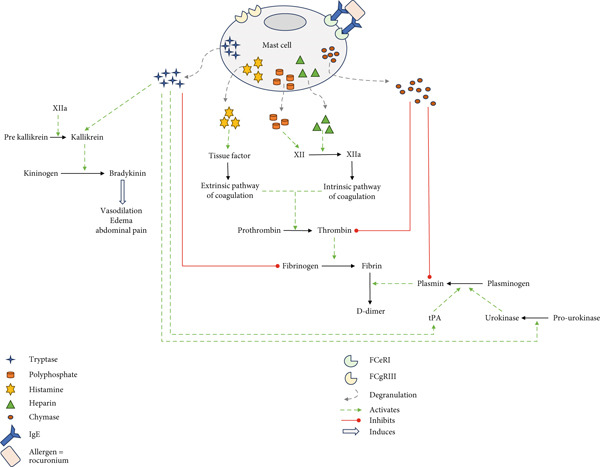
Influence of mast cell degranulation on the coagulation pathway.

MCs, multipotent effector cells of the immune system, reside in the connective tissues of nearly all organ systems around blood capillaries, to respond to aggression [13, 18]. They play a critical role in anaphylaxis, wherein Ig‐E‐mediated MC degranulation triggers the release of vasoactive mediators such as histamine, serotonin, heparin, chymase, PAF, and tryptase (Figure 2). The release of tryptase by MCs may lead to profound coagulopathy [13]. Normally, the circulating MC tryptase concentration remains close to zero. However, following anaphylaxis, tryptase levels increase and reach a peak about an hour later, gradually decreasing with a half‐life of approximately two hours. A tryptase level exceeding 10 *μ*g/L is considered indicative of anaphylaxis. Tryptase‐negative anaphylaxis has been described without elevated tryptase or clear MC activation. The underlying mechanisms remain incompletely understood, and basophils may be involved, but are not necessarily the main effector cells. Without a skin test, it becomes challenging to distinguish between a non‐IgE‐mediated anaphylaxis and an anaphylactic reaction [13].

MC degranulation (and the release of mediators) can influence the coagulation pathway, the kallikrein–kinin system, and fibrinolysis (Figure 1). Among these mediators, tryptase holds a prominent position, as studies have demonstrated its ability to activate tissue and urokinase‐type plasminogen activators, thereby promoting plasmin production [12]. This cascade leads to hyperfibrinolysis, resulting in an increase in the breakdown products of fibrinogen. Moreover, tryptase specifically cleaves the *α* and *β* chains of fibrinogen, effectively eliminating two crucial sites on the *β* chain of fibrinogen: the thrombin cleavage site and a critical polymerization site. Consequently, the formation of clots initiated by thrombin is inhibited. Simultaneously, the coagulation pathway can be impeded by the release of heparin from MCs. The contact system—comprising factor XII (FXII), prekallikrein, and high molecular‐weight kininogen—is activated, likely in response to heparin release from MCs. This heparin provides a negatively charged surface that promotes FXII activation, triggering the kallikrein‐kinin cascade and the generation of bradykinin (BK). BK induces peripheral vasodilation, increased vascular permeability, angioedema, hypotension, and abdominal pain. Moreover, although less efficiently than tPA or uPA, FXII can activate plasminogen, and conversely, plasmin can activate FXII, highlighting the bidirectional interactions between the fibrinolytic system and the contact system [7].

The degradation of fibrinogen and other coagulation factors theoretically induces a potentially hemorrhagic state [17]. The low prevalence of bleeding may be attributed to the activation of FXII by the kallikrein–kinin system, which itself is activated by heparin, tryptases, and polyphosphates released by MCs [18].

Although cases of coagulation disorders resulting from anaphylaxis are rare, recognizing their occurrence is crucial. Intraoperative anaphylaxis is often associated with curares, with rocuronium being one of the most likely to induce such a reaction, with a reported incidence of 1 case of anaphylaxis for every 2499 new exposures [19]. As curares are increasingly used in acute medicine, it is expected that similar cases will be reported more frequently. Therefore, it is essential to raise awareness among anesthetists, emergency physicians, and resuscitators about the risk of DIC, hyperfibrinogenolysis, and hypofibrinogenemia during anaphylaxis and encourage them to test for coagulation abnormalities in the acute phase of an anaphylactic reaction. Accurately assessing coagulation disorders requires a combination of static blood tests specific to various coagulation pathways (INR, PTT, and fibrinogen) along with viscoelastic coagulation tests.

Despite clear signs of biological coagulopathy, a hemorrhagic tendency was rarely reported in case reports. Self‐resolution often occurs within a few hours, suggesting that fibrinolysis associated with anaphylaxis is a self‐limiting process. Due to the rarity of the situation, management is not standardized. Various doses of tranexamic acid (ranging from 200 mg to 1 g) and fresh frozen plasma (FFP) have been proposed. However, it should be noted that tranexamic acid and FFP are not without side effects. Therefore, their administration should be guided by clinical considerations rather than being systematic and solely based on viscoelastic coagulation tests. The optimal dose of antifibrinolytics or coagulation factors has yet to be determined [13].

## 4. Conclusion

This clinical case highlights the development of hypofibrinogenemia as a secondary effect of various mediators, such as tryptases, released during MC degranulation in severe anaphylaxis. During anesthesia, curares, including rocuronium, can induce anaphylaxis and, consequently, coagulopathy. With the increasing use of curares in the routine practice of anesthetists, resuscitators, and emergency physicians, it becomes crucial to raise awareness among these medical professionals regarding the risk of coagulopathy in anaphylactic shock and its detection through viscoelastic coagulation tests.

## Ethics Statement

Ethical approval was not required for this case report in accordance with local or national guidelines. Nevertheless, written informed consent was obtained from the patient for publishing the details of his medical case.

## Conflicts of Interest

The authors declare no conflicts of interest.

## Author Contributions

Data collection: Cedric Hermans and Caroline van de Wyngaert; writing—original draft: Caroline van de Wyngaert and Michael Iarossi; writing—review and editing: Véronique Hamoir, Geoffroy Vanderweerden, Cedric Hermans, Caroline van de Wyngaert, and Michael Iarossi. Michael Iarossi and Caroline van de Wyngaert contributed equally to the conduction of the study and the writing of the manuscript.

## Funding

No funding was received for this manuscript.

## Data Availability

All data generated or analyzed during this study were included in this article. Further enquiries can be directed to the corresponding author.
